# Long-Term Outcomes of the Minimally Invasive Ponto Surgery vs. Linear Incision Technique With Soft Tissue Preservation for Installation of Percutaneous Bone Conduction Devices

**DOI:** 10.3389/fneur.2021.632987

**Published:** 2021-02-24

**Authors:** Ruben M. Strijbos, Louise V. Straatman, Tim G. A. Calon, Martin L. Johansson, Arthur J. G. de Bruijn, Herbert van den Berge, Mariette Wagenaar, Edwin Eichhorn, Miranda Janssen, Sofia Jonhede, Joost van Tongeren, Marcus Holmberg, Robert Stokroos

**Affiliations:** ^1^Department of Otorhinolaryngology and Head and Neck Surgery, University Medical Centre Utrecht, Utrecht, Netherlands; ^2^University Medical Centre Utrecht Brain Centre, University of Utrecht, Utrecht, Netherlands; ^3^Department of Biomaterials, Institute of Clinical Sciences, Sahlgrenska Academy, University of Gothenburg, Gothenburg, Sweden; ^4^Oticon Medical AB, Askim, Sweden; ^5^Department of Otorhinolaryngology, Ziekenhuisgroep Twente, Almelo, Netherlands; ^6^Department of Otorhinolaryngology, Medisch Centrum Leeuwarden, Leeuwarden, Netherlands; ^7^Department of Otorhinolaryngology and Head and Neck Surgery, Maastricht University Medical Centre, Maastricht, Netherlands; ^8^Department of Methodology and Statistics, Care and Public Health Research Institute, Maastricht University, Maastricht, Netherlands

**Keywords:** hearing loss, bone conduction device (BCD), surgical technique, minimally invasive ponto surgery, MIPS, tissue preservation, surgical outcomes, soft tissue reactions

## Abstract

**Objective:** Comparing the surgical outcomes of the Minimally Invasive Ponto Surgery (MIPS) technique with the linear incision technique with soft tissue preservation (LITT-P) for bone conduction devices after a follow-up of 22 months.

**Methods:** In this multicenter randomized controlled trial, there was the inclusion of 64 adult patients eligible for unilateral surgery. There was 1:1 randomization to the MIPS (test) or the LITT-P (control) group. The primary outcome was an (adverse) soft tissue reaction. Secondary outcomes were pain, loss of sensibility, soft tissue height/overgrowth, skin sagging, implant loss, Implant Stability Quotient measurements, cosmetic scores, and quality of life questionnaires.

**Results:** Sixty-three subjects were analyzed in the intention-to-treat population. No differences were found in the presence of (adverse) soft tissue reactions during complete follow-up. Also, there were no differences in pain, wound dehiscence, skin level, soft tissue overgrowth, and overall quality of life. Loss of sensibility (until 3-month post-surgery), cosmetic scores, and skin sagging outcomes were better in the MIPS group. The Implant Stability Quotient was higher after the LITT-P for different abutment lengths at various points of follow-up. Implant extrusion was nonsignificantly higher after the MIPS (15.2%) compared with LITT-P (3.3%).

**Conclusion:** The long-term results show favorable outcomes for both techniques. The MIPS is a promising technique with some benefits over the LITT-P. Concerns regarding nonsignificantly higher implant loss may be overcome with future developments and research.

**Clinical Trial Registration:** www.ClinicalTrials.gov, identifier: NCT02438618.

## Introduction

Bone conduction devices (BCDs) have become increasingly important in hearing rehabilitation. The BCD comprises a retro-auricular implant in the skull to which a sound processor is connected *via* a skin-penetrating abutment (1). Treatment with a BCD is indicated in patients with uni- and bilateral conductive or mixed hearing loss, with intolerance or inability to wear conventional hearing aids (1–3), or in patients with single-sided deafness (4). BCD surgery is considered a safe procedure with a low risk of complications (5). Soft tissue problems, including peri-abutment inflammation [as graded by the Holgers Index (6)], skin thickening, and tissue overgrowth, do occur. Other complications are numbness and pain at the implant site, pain, postoperative wound dehiscence or skin necrosis, and implant extrusion (5, 7). Therefore, further development of implantation techniques and improvements of the BCD implants would be beneficial.

Besides variations in implant and abutment design, for example, the hydroxyapatite layer of the BIA400 from Cochlear (Mölnlycke, Sweden) (8, 9) and the smooth titanium surface of the Ponto Wide from Oticon Medical AB (Askim, Sweden) (10, 11), there have been advances in surgical techniques. Initially, subcutaneous tissue was removed in an attempt to reduce friction skin movements around the abutment. Different surgical techniques were introduced, including the free retro-auricular full-thickness skin graft, pedicled grafts, the dermatome technique, and finally leading up to the universally adopted linear incision technique with tissue reduction (LITT-R) (12, 13). Of these surgical techniques, the LITT-R gained popularity because it leads to fewer complications and is a straightforward procedure (13–15). A different approach to achieve reduced soft tissue reaction is the usage of transcutaneous BCDs. These are abutment-free, which might reduce soft tissue problems (16). However, audiological outcomes are often less favorable due to attenuation of (sound) vibration by the soft tissue (17, 18). For the percutaneous devices, in the context of surgical techniques, the linear incision technique with soft tissue preservation (LITT-P) was developed based on this principle of less invasive surgery. Studies did show improved outcomes, including more favorable cosmetic results, less numbness, and shorter surgical time (19–25). The vast majority of the previous studies evaluating the outcome of this technique suggested similar or less (adverse) soft tissue reactions in comparison with the LITT-R (7, 24–28).

Recently, to further reduce soft tissue reactions, the surgical procedure was simplified to a minimally invasive, so-called punch-only technique (29–32). These procedures with a punch-hole only should theoretically result in less soft tissue trauma. Over the last years, several surgeons performed this principle of punch-only technique and described improved cosmetic results and shorter surgical procedure time without observing more soft tissue problems in comparison with the dermatome or linear incision technique (29–32). A standardized approach for the technique was lacking. This was the reason for Oticon Medical AB (Askim, Sweden) to introduce a new standardized punch-only technique, including a surgical kit: the Minimally Invasive Ponto Surgery (MIPS) (33).

The short-term results of a multicenter evaluation using the MIPS were encouraging with minimal intraoperative complications (e.g., only one case of a cerebrospinal fluid leak in 77 implants). Also, the outcomes regarding soft tissue reactions (5.0% adverse soft tissue reactions recorded in 160 visits) and implant survival rates (96.1% at 20 weeks) were promising (33). Moreover, another direct cost comparison study demonstrated a reduction in cost with the MIPS in comparison with the linear incision approach (34). In a multicenter randomized controlled trial, Calon et al. (35) compared the MIPS technique with the LITT-P. After 3 months of follow-up, the MIPS resulted in significantly less skin sagging and numbness of the skin. Furthermore, there was a significant reduction of surgical time and an improvement in cosmetic outcomes. There were no significant differences in soft tissue inflammation (Holgers score ≥ 2) between the procedures. Nonetheless, a nonsignificant increase in implant extrusion rate was found when using the MIPS technique (35). Besides these encouraging short-term results, however, there is only one small prospective cohort study of Sardiwalla et al. (36) with a longer follow-up (minimal 12 months). This study concluded device stability and patient satisfaction with the MIPS procedure (36).

These findings warrant exploration of the long-term results of the MIPS technique. The current study will compare the surgical outcomes of the MIPS procedure with the LITT-P after a follow-up of 22 months. To our knowledge, this is the first well-designed, multicenter randomized controlled study that will present and discuss the long-term results of the MIPS technique.

## Materials and Methods

### Study Design and Subjects

This study is a multicenter randomized controlled trial in the Netherlands (Maastricht University Medical Centre, Ziekenhuisgroep Twente, and Medisch Centrum Leeuwarden). The protocol of this study was published previously (37), as well as the surgical outcomes after 3 months of follow-up (35).

The inclusion criteria were eligibility for unilateral BCD surgery (38) in combination with an adult age (≥18 years). Patients with a history of immunosuppressive disease and/or systemic immunosuppressive medication, relevant dermatological disease, bilateral BCD placement, and participation in other studies were excluded. In case of the preoperative absence of a suitable implantation site for a 4.0-mm implant or insufficient bone quality, the subject was regarded as early termination and excluded from the study. All enrolled subjects were randomized in each research center independently in a 1:1 ratio stratified for sex. The test group was the MIPS technique, and the control cohort was the LITT-P.

### Surgical Technique and Post-surgery Protocol

All otorhinolaryngologists were experienced in the LITT-P procedure and had instruction and training in the MIPS procedure. Depending on patient preferences, local, or general anesthesia was administered. Measurement of the skin thickness (before application of local anesthesia) was used to determine the abutment length. In both techniques, a Ponto-wide 4-mm implant with a premounted abutment (9, 12, or 14 mm) was installed using an insertion torque setting of 40–50 Ncm (Oticon Medical, Askim, Sweden).

The procedure of the LITT-P (control group) consists of a longitudinal incision, which is located to the ear canal posterosuperior. The implant is placed in the temporal bone after mobilizing the skin and subcutaneous tissue and exposure of the periosteum. The skin is punched outside the incision line, and the abutment is guided through the punch hole. For the MIPS technique (test group), an incision was created with a 5-mm punch with the removal of the remaining soft tissue and periosteum in the punch hole. The implant positioning is similar to the LITT-P. A cannula is inserted at the surgical site, and, after that, the hole is created with the cannula guide drill followed by the cannula widening drill. Then, the implant with abutment is installed, assisted with the insertion indicator. There is an extensive deliberation of both surgical techniques, including step-by-step illustrations in the study protocol (37).

The assessment of the patients was at baseline, surgery, 9 days postoperative, 3 weeks post-surgery, and after 3, 12, and 22 months of follow-up. Patients or physicians could initiate extra visits in case of complications, other problems, or individual requests. The different outcome measures were registered accurately during all these follow-up appointments at different points in time.

### Outcome Measures

The primary outcome is the incidence of an adverse soft tissue reaction (Holgers index ≥ 2) between surgery and 22 months of follow-up. Secondary outcomes are pain directly around the abutment or related to the implant, loss of sensibility of the skin, wound dehiscence, soft tissue height/overgrowth, presence of skin sagging, implant loss, and implant stability quotient (ISQ) measurements. The cosmetic result score (graded using a 10-point scale) is measured after 3-, 12-, and 22-months follow-up. The tertiary outcomes consist of quality of life questionnaires: the Health Utilities Index Mark III (HUI-III), Abbreviated Profile of Hearing Aid Benefit (APHAB), and ICEpop CAPability measure for Adults (ICECAP-A). These questionnaires were executed at baseline consultation and after 12 and 22 months or any visit with an adverse soft tissue reaction. Also, complications, adverse events, and serious adverse events were recorded.

### Statistical Analysis

The data analysis was conducted by Statistika Konsultgruppen (Gothenburg, Sweden). An intention-to-treat (ITT) and per-protocol (PP) population analysis was performed for all surgical outcomes. The level of statistical significance applied was *p* = 0.05.

The statistical test for the primary outcome adverse soft tissue reactions was a chi-square test and Fisher's exact test. Additionally, a Mantel–Haenszel chi-square test was executed to identify differences in Holgers scores. For the secondary and tertiary outcomes, the comparison between the cohorts in the presence of sensibility loss, skin sagging, wound dehiscence, and soft tissue overgrowth (i.e., the number of abutment replacements and revision surgeries) was performed with a Fisher's exact test. The analysis of the endpoints pain, area of sensibility loss, skin level, ISQ measures (high and low), cosmetic results, and all quality of life questionnaires was with a Mann–Whitney *U*-test. A Kaplan–Meier curve was created for the implant extrusion, and a log-rank test was executed to compare both groups.

### Ethics

This study was performed in accordance with ISO 14155:2011 and the Declaration of Helsinki. There was approval by the ethics committee at Maastricht University Medical Centre+ (NL500720.068.14), Medisch Centrum Leeuwarden, and Ziekenhuisgroep Twente. Also, there was registration in ClinicalTrials.gov NCT02438618. The participation of subjects was voluntary, and all subjects provided written informed consent. The study is sponsored by Oticon Medical AB (Askim, Sweden). The investigators had full access to all data. Monitoring was performed independently.

## Results

### Baseline Characteristics

**Figure 1:**
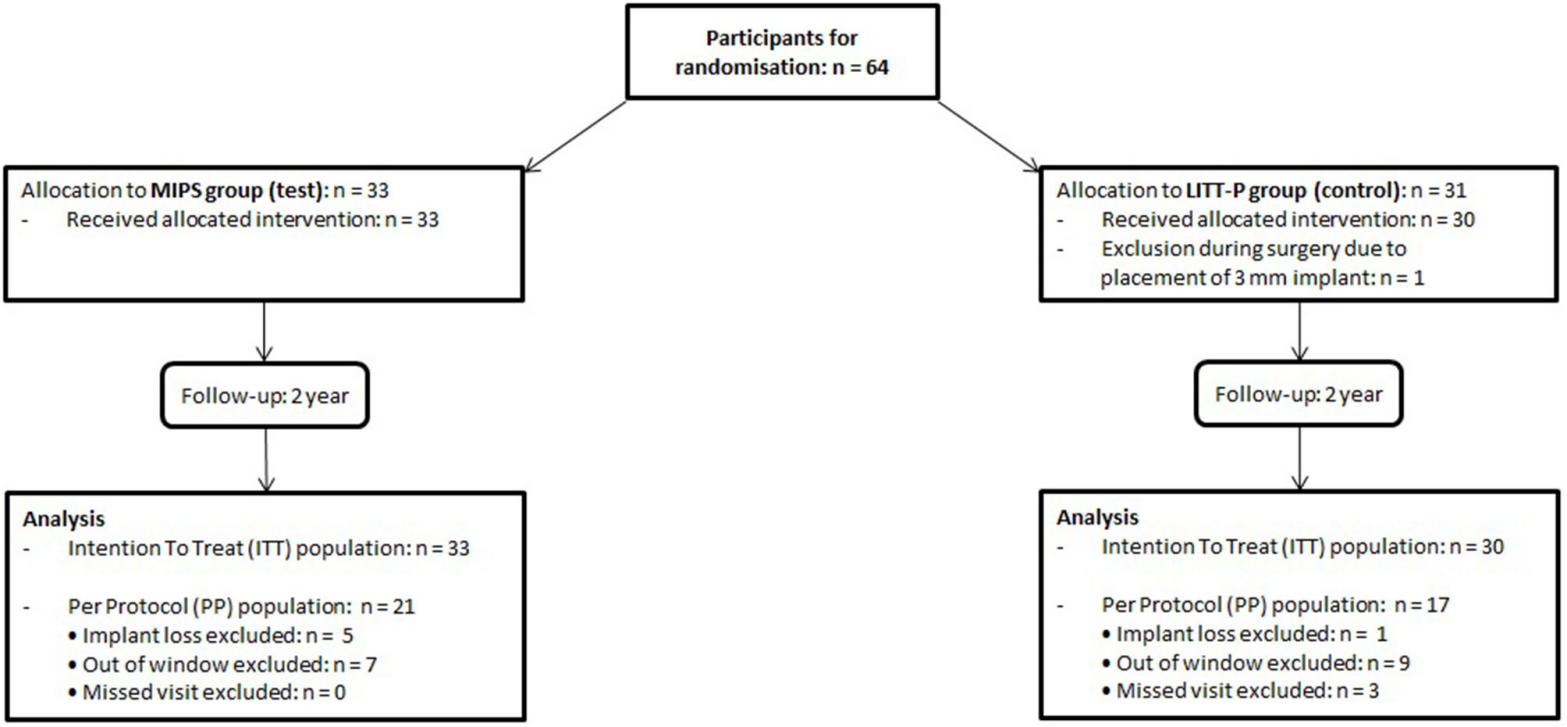
Subject flow chart.

Sixty-four participants were included between December 2014 and August 2016. Thirty-three subjects were randomized to the test cohort (52%) and 31 to the control cohort (48%). There was the exclusion of one patient during surgery because of the placement of a 3-mm implant. This resulted in 63 subjects being analyzed in the ITT analysis. Due to implant loss and protocol deviations (mainly visits out of the window but also missed standard visits), 25 subjects were excluded from the ITT group, which resulted in a total PP population of 38 participants (Figure 1). Baseline characteristics were comparable between the groups (for both ITT and PP population, see Table 1, Supplementary Table 1).

**Table 1:** Baseline characteristics intention-to-treat population.

**Baseline characteristics**	**MIPS (*n* = 33)**	**LITT-P (*n* = 30)**	***p*-value**
**Age (years)**			
Mean (SD), 95%–CI	50.3 (16.3), 44, 5; 56.1	51.9 (16.1), 45.9; 57.9	0.52
Median (Min–Max)	51.0 (19.0–80.0)	58.5 (21.0–75.0)	
**Gender**			
Male	12 (36.4%)	11 (36.7%)	1.00
Female	21 (63.6%)	19 (63.3%)	
**Type of hearing loss**			
Acquired conductive/mixed hearing loss	26 (78.8%)	25 (83.3%)	0.62
Single sided deafness	6 (18.2%)	5 (16.7%)	
Congenital conductive hearing loss	1 (3.0%)	0 (0.0%)	
**Smoking**			
Yes	7 (21.2%)	8 (26.7%)	0.83
No	26 (78.8%)	22 (73.3%)	
**Body Mass Index (BMI; kg/m2)**			
Mean (SD), 95%–CI	27.4 (6.4), 25.2; 29.7	28.4 (5.7), 26.2; 30.5	0.36
Median (Min–Max)	27.2 (19.6–44.4)	26.7 (20.6–45.0)	
**Ethnicity**			
Caucasian	33 (100.0%)	30 (100.0%)	
Implantation site			
Right	17 (51.5%)	13 (43.3%)	0.69
Left	16 (48.5%)	17 (56.7%)	
**Abutment length**			
9	21 (63.6%)	13 (43.3%)	0.11
12	10 (30.3%)	16 (53.3%)	0.064
14	2 (6.1%)	1 (3.3%)	

### Primary Outcome

**Figure 2:**
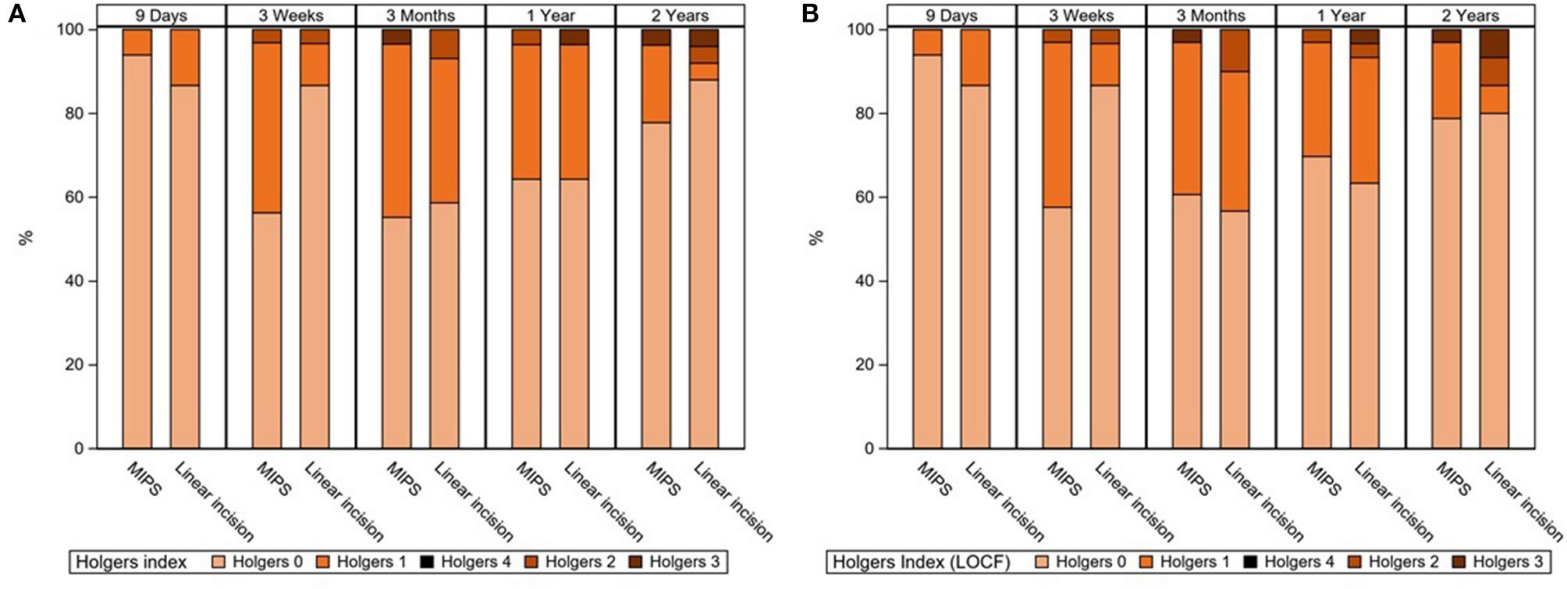
**(A)** Stacked bar chart for the highest observed Holgers Index scores during standard follow-up visits. In the right-sided figure **(B)**, missing data were corrected with the last observation carried forward technique. Last visit (“2 years”) was at 22 months of follow-up.

There was no difference in adverse soft tissue reaction (Holgers ≥ 2) during the 22-month follow-up between the MIPS and LITT-P groups in either the ITT or the PP populations (Table 2, Supplementary Table 2, Figure 2, Supplementary Figure 1). Sensitivity analyses did not reveal any significant difference either. Moreover, no difference was found in the maximum Holgers index between the groups.

**Table 2:** Primary and secondary outcomes (ITT population).

		**MIPS (*n* = 33)**	**LITT-P (*n* = 30)**	***p*-value**
**Primary outcome: (adverse) soft tissue reactions**				
Adverse soft tissue reaction (Holgers ≥ 2) from surgery to 22 months		8 (24.2%)	11 (36.7%)	0.28
Adverse soft tissue reaction (Holgers ≥ 2) from surgery to 22 months (with the Fisher's Exact Test)		8 (24.2%)	11 (36.7%)	0.42
Adverse soft tissue reaction (Holgers ≥ 2) from surgery to 22 months (Sensitivity analysis: highest observed Holgers score plus one)		8 (24.2%)	11 (36.7%)	0.28
Adverse soft tissue reaction (Holgers ≥ 2) from surgery to 22 months (Sensitivity analysis: all observed implant losses have experienced a Holgers Index score of four)		12 (36.4%)	11 (36.7%)	0.98
Maximum Holgers Index at standard and extra visits				
0 No irritation		11 (33.3%)	6 (20.0%)	0.38
1 Slight redness		14 (42.4%)	13 (43.3%)	
2 Red and slightly moist tissue		4 (12.1%)	9 (30.0%)	
3 Reddish and moist tissue, sometimes granulation formation		4 (12.1%)	2 (6.7%)	
4 Profound signs of infection resulting in implant removal		0 (0.0%)	0 (0.0%)	
**Secondary outcome: pain**				
Pain around the implant				
9 days		1.39 (1.87, 0.73; 2.06) 0.00 (0.00–6.00) *n* = 33	1.97 (2.61, 0.99;2.94) 1.00 (0.00–8.00) *n* = 30	0.50
3 weeks		0.938 (1.216, 0.499; 1.376) 0.00 (0.00–4.00) *n* = 32	1.000 (1.619, 0.396; 1.604) 0.00 (0.00–6.00) n= 30	0.67
3 months		1.38 (2.23, 0.53; 2.23) 0.00 (0, 00–8.00) n = 29	1.17 (2.04, 0.40; 1.95) 0.00 (0.00–7.00) *n* = 29	0.54
12 months		0.778 (1.739, 0.090; 1.466) 0.00 (0.00–6.00) n = 27	1.54 (2.43, 0.59; 2.48) 0.00 (0.00–8.00) *n* = 28	0.23
22 months		0.889 (2.190, 0.023; 1.755) 0.00 (0.00–9.00) *n* = 27	0.680 (1.492, 0.064; 1.296) 0.00 (0.00–5.00) *n* = 25	0.97
Radiating pain from the implant				
9 days		0.606 (1.657, 0.018; 1.194) 0.00 (0.00–7.00) *n* = 33	0.500 (1.570,−0.086; 1.086) 0.00 (0.00–8.00) *n* = 30	0.95
3 weeks		0.563 (1.390, 0.061; 1.064) 0.00 (0.00–5.00) *n* = 32	0.433 (1.357,−0.073; 0.940) 0.00 (0.00–5.00) *n* = 30	0.39
3 months		0.759 (1.864, 0.050; 1.468) 0.00 (0.00–6.00) *n* = 29	0.759 (1.883, 0.042; 1.475) 0.00 (0.00–7.00) *n* = 29	0.77
12 months		0.571 (1.814,−0.132; 1.275) 0.00 (0.00–8.00) *n* = 28	0.464 (1.453,−0.099; 1.028) 0.00 (0.00–6.00) *n* = 28	0.75
22 months		0.481 (1.868,−0.258; 1.221) 0.00 (0.00–9.00) *n* = 27	0.200 (0.707,−0.092; 0.492) 0.00 (0.00–3.00) *n* = 25	1.00
Headache related to the BCD				
9 days		0.424 (1.393,−0.070; 0.918) 0.00 (0.00–7.00) *n* = 33	1.30 (2.39, 0.41; 2.19) 0.00 (0.00–8.00) *n* = 30	0.077
3 weeks		0.375 (1.476,−0.157; 0.907) 0.00 (0.00–6.00) *n* = 32	0.300 (1.317,−0.192; 0.792) 0.00 (0.00–7.00) *n* = 30	0.96
3 months		0.793 (2.094,−0.003; 1.590) 0.00 (0.00–8.00) *n* = 29	0.241 (0.830,−0.075; 0557) 0.00 (0.00–4.00) *n* = 29	0.59
12 months		0.464 (1.478,−0.109; 1.037) 0.00 (0.00–6.00) *n* = 28	0.00 (0.00, 0.00; 0.00) 0.00 (0.00–0.00) *n* = 28	0.081
22 months		0.296 (1.540,−0.313; 0.905) 0.00 (0.00–8.00) *n* = 27	0.280 (1.400,−0.298; 0.858) 0.00 (0.00–7.00) *n* = 25	1.00
**Secondary outcome: sensibility**				
Area loss of sensibility				
9 days		2.70 (6.13, 0.52; 4.87) 0.00 (0.00–25.00) *n* = 33	13.5 (21.0, 5.6; 21.3) 4.5 (0.00–100.0) *n* = 30	**0.005**
3 weeks		0.375 (1.040, 0.000; 0.750) 0.00 (0.00–5.00) *n* = 32	8.23 (17.25, 1.79; 14.68) 0.00 (0.00–70.00) *n* = 30	**0.013**
3 months		0.138 (0.516,−0.058; 0.334) 0.00 (0.00–2.00) *n* = 29	5.79 (13.75, 0.56; 11.02) 0.00 (0.00–60.00) *n* = 29	**0.0076**
12 months		0.679 (2.374,−0.242; 1.599) 0.00 (0.00–10.00) *n* = 28	2.93 (10.18,−1.10; 6.95) 0.00 (0.00–50.00) *n* = 27	0.60
22 months		1.000 (4.472,−1.093; 3.093) 0.00 (0.00–20.00) *n* = 20	0.222 (0.943,−0.247; 0.691) 0.00 (0.00–4, 00) *n* = 18	1.00
Presence of loss of sensibility?				
9 days	No	24 (72.7%)	13 (43.3%)	**0.034**
	Yes	9 (27.3%)	17 (56.7%)	
3 weeks	No	27 (84.4%)	18 (60.0%)	0.061
	Yes	5 (15.6%)	12 (40.0%)	
3 months	No	27 (93.1%)	19 (65.5%)	**0.021**
	Yes	2 (6.9%)	10 (34.5%)	
12 months	No	25 (89.3%)	23 (85.2%)	0.96
	Yes	3 (10.7%)	4 (14.8%)	
22 months	No	19 (95.0%)	17 (94.4%)	1.00
	Yes	1 (5.0%)	1 (5.6%)	
**Secondary outcome: cosmetic appearance**				
Natural skin position				
3 months		2.72 (1.10, 2.31; 3.14) 3.00 (1.00–5.00) *n* = 29	3.48 (1.38, 2.96; 4.01) 3.00 (1.00–6.00) *n* = 29	**0.025**
12 months		2.07 (1.15, 1.62; 2.52) 2.00 (1.00–5.00) *n* = 28	2.82 (1.33, 2.30; 3.34) 3.00 (1.00–5.00) *n* = 28	**0.026**
22 months		2.12 (1.72, 1.41; 2.83) 2.00 (1.00–8.00) *n* = 25	2.23 (1.54, 1.54; 2.91) 2.00 (1.00–7.00) *n* = 22	0.46
Extent of baldness				
3 months		2.24 (0.79, 1.94; 2.54) 2.00 (1.00–4.00) *n* = 29	3.62 (1.35, 3.11; 4.13) 4.00 (1.00–6.00) *n* = 29	** < .0001**
12 months		1.93 (0.94, 1.56; 2.29) 2.00 (1.00–4.00) *n* = 28	2.81 (1.55, 2.20; 3.43) 3.00 (1.00–6.00) *n* = 27	**0.038**
22 months		1.92 (1.75, 1.20; 2.64) 1.00 (1.00–9.00) *n* = 25	1.95 (1.00, 1.51; 2.40) 2.00 (1.00–4.00) *n* = 22	0.30
Scarring				
3 months		2.41 (0.95, 2.05; 2.77) 2.00 (1.00–5.00) *n* = 29	4.48 (1.79, 3.80; 5.16) 5.00 (1.00–7.00) n =29	** < .0001**
12 months		2.11 (1.20, 1.64; 2.57) 2.00 (1.00–5.00) *n* = 28	3.64 (1.79, 2.95; 4.34) 4.00 (1.00–7.00) *n* = 28	**0.001**
22 months		2.28 (1.67, 1.59; 2.97) 2.00 (1.00–9.00) *n* = 25	2.23 (1.07, 1.75; 2.70) 2.00 (1.00–5.00) *n* = 22	0.66
Skin color				
3 months		3.17 (1.23, 2.71; 3.64) 3.00 (1.00–7.00) *n* = 29	3.86 (1.27, 3.38; 4.35) 4.00 (1.00–6.00) *n* = 29	**0.020**
12 months		2.36 (0.99, 1.97; 2.74) 2.00 (1.00–4.00) *n* = 28	3.25 (1.40, 2.71; 3.79) 4.00 (1.00–6.00) *n* = 28	**0.013**
22 months		2.24 (1.79, 1.50; 2.98) 2.00 (1.00–9.00) *n* = 25	2.23 (1.23, 1.68; 2.77) 2.00 (1.00–5.00) *n* = 22	0.61
Indentation				
3 months		2.34 (1.01, 1.96; 2.73) 2.00 (1.00–5.00) *n* = 29	4.00 (1.63, 3.38; 4.62) 4.00 (1.00–7.00) n =29	** < .0001**
12 months		2.26 (1.26, 1.76; 2.76) 2.00 (1.00–5.00) *n* = 27	3.33 (1.80, 2.62; 4.04) 3.00 (1.00–7.00) *n* = 27	**0.024**
22 months		2.50 (2.40, 1.49; 3.51) 1.00 (1.00–9.00) *n* = 24	2.23 (1.69, 1.48; 2.98) 2.00 (1.00–7.00) *n* = 22	0.85
Overall cosmetic score				
3 months		8.45 (0.74, 8.17; 8.73) 8.00 (7.00–10.00) *n* = 29	7.17 (1.20, 6.72; 7.63) 7.00 (6.00–10.00) *n* = 28	** < .0001**
12 months		8.14 (2.21, 7.29; 9.00) 9.00 (1.00–10.00) *n* = 28	7.50 (1.75, 6.82; 8.18) 7.00 (1.00–10.00) *n* = 28	**0.014**
22 months		7.96 (1.95, 7.16; 8.76) 9.00 (2.00–10.00) *n* = 25	7.68 (1.70, 6.93; 8.44) 8.00 (2.00–10.00) *n* = 22	0.20
Satisfaction with result without processor				
3 months		8.42 (1.47, 7.83; 9.02) 9.00 (4.00–10.00) *n* = 26	8.61 (1.29, 8.11; 9.11) 9.00 (6.00–10.00) *n* = 28	0.75
12 months		8.20 (1.38, 7.63; 8.77) 9.00 (5.00–10.00) *n* = 25	8.64 (1.25, 8.16; 9.13) 8.50 (6.00–10.00) *n* = 28	0.30
22 months		8.25 (1.96, 7.42; 9.08) 9.00 (3.00–10.00) *n* = 24	8.41 (1.53, 7.73; 9.09) 8.50 (3.00–10.00) *n* = 22	0.93
Satisfaction with result with processor				
3 months		7.41 (2.58, 6.39; 8.43) 8.00 (1.00–10.00) *n* = 27	7.89 (1.83, 7.18; 8.60) 8.00 (3.00–10.00) *n* = 28	0.73
12 months		7.52 (2.54, 6.47; 8.57) 8.00 (1.00–10.00) *n* = 25	7.96 (2.05, 7.17; 8.76) 8.00 (1.00–10.00) *n* = 28	0.72
22 months		7.39 (2.52, 6.30; 8.48) 8.00 (2.00–10.00) *n* = 23	7.90 (1.58, 7.19; 8.62) 8.00 (4.00–10.00) *n* = 21	0.80
**Secondary outcome: soft tissue**				
Mean skin level				
9 days		4.73 (1.66, 4.14; 5.33) 5.00 (0.00–7.25) *n* = 32	5.53 (1.15, 5.09; 5.96) 5.50 (3.00–7.25) *n* = 29	0.052
3 weeks		4.54 (1.58, 3.97; 5.11) 5.00 (0.00–7.00) *n* = 32	4.95 (1.05, 4.56; 5.34) 5.00 (3.00–7.00) *n* = 30	0.33
3 months		5.02 (1.42, 4.48; 5.56) 5.00 (2.75–8.00) *n* = 29	5.08 (1.04, 4.68; 5.47) 5.00 (2.50- 7.50) *n* = 29	0.64
12 months		5.10 (1.76, 4.42; 5.78) 5.00 (1.00–8.00) *n* = 28	5.52 (1.16, 5.06; 5.98) 5.50 (3.50–8.00) *n* = 27	0.43
22 months		5.04 (1.84, 4.30; 5.78) 4.75 (1.00–9.00) *n* = 26	5.26 (1.32, 4.71; 5.81) 5.00 (2.50–8.50) n =25	0.52
Skin sagging in any quadrant				
9 days		7 (21.2%)	15 (51.7%)	**0.024**
3 weeks		11 (34.4%)	21 (70.0%)	**0.010**
3 months		8 (27.6%)	20 (71.4%)	**0.002**
12 months		9 (32.1%)	16 (57.1%)	0.11
22 months		6 (23.1%)	13 (52.0%)	0.064
Wound dehiscence				
9 days		16 (48.5%)	22 (33.3%)	0.078
3 weeks		4 (12.5%)	4 (13.3%)	1.00
3 months		1 (3.3%)	0 (0.0%)	1.00
12 months		1 (3.6%)	0 (0.0%)	1.00
22 months		0 (0.0%)	0 (0.0%)	1.00
Soft tissue overgrowth				
Abutment changes		3 (9.1%)	3 (10.0%)	1.00
Revision surgery		2 (6.1%)	1 (3.3%)	1.00
**Secondary outcome: implant extrusion**				
Implant loss		5 (15.2%)	1 (3.3%)	0.12

Continuous variables are presented as mean (SD, 95% CI) and median (min-max) with n. A significant p-value (p < 0.05) is showed in bold. Pain is graded on a 10-point scale with a scale of 0 representing absence of pain to 10 representing worst pain. In cases of missing values, a correction was calculated with last observation carried forward technique, which did either not show significant differences. Area of sensibility loss is registered as most outward diameter from abutment (in millimeters). Cosmetic observed variables are rated as 1 being no difference with healthy contralateral site and with 10 being most negative difference with health situation. In contrast, overall cosmetic score and patient satisfaction will be rated with 10 being best cosmetic result. The skin level is measured as distance between top of abutment to skin in four quadrants (in millimeters).

### Secondary Outcomes

#### Pain and Sensibility

During a complete follow-up of 22 months, there were no significant differences in the presence of pain around the implant, radiating pain, and/or headache related to the BCD. At 3 weeks and subsequent follow-up, the mean pain scores were <2 of 10. The loss of sensibility was significantly less in the MIPS cohort in comparison with the LITT-P group for the follow-up visits until 3 months after surgery in the ITT population. No differences in loss of sensibility were found at 12- and 22-month follow-ups for either the ITT or PP population (Table 2, Supplementary Table 2).

#### Cosmetic Outcomes

The outcomes of natural skin position, the extent of baldness, scarring, skin color, indentation, and overall cosmetic score (as assessed by the surgeon and subject) were significantly better in the MIPS group at 3 months and 1-year follow-up (Table 2, Supplementary Table 2). There were no differences between the surgical techniques at 22 months of follow-up except for the overall cosmetic score in the PP population (*p* < 0.01). The patient satisfaction in cosmetics with the result with (and without) processor attached did not differ between the two groups, and all scores were generally favorable during complete follow-up. An overview of the cosmetic results is presented in Table 2, Supplementary Table 2.

#### Soft Tissue Outcomes

Skin sagging was generally significantly more present in patients who underwent the LITT-P compared with MIPS at different time points during the follow-up of 22 months (in the first 3 months for the ITT population and during the complete follow-up in the PP population) (Table 2, Supplementary Table 2). The mean skin level, measured as the distance between the top of the abutment to the skin in four quadrants, did not significantly differ between the two techniques during the follow-up. Also, the incidence of soft tissue overgrowth requiring abutment change or revision surgery was rare and did not differ between the groups. Abutment change was necessary for two patients (both in the LITT-P cohort), whereas four abutments were electively removed (one patient in the LITT-P cohort and the other three patients in the MIPS cohort). Revision surgery was performed in two patients in the MIPS group and one patient in the LITT-P group. The presence of wound dehiscence did not differ between the groups during the follow-up. Table 2, Supplementary Table 2 illustrates these soft tissue outcomes.

#### Implant Extrusion

A total of six implants (9.5%) were extruded, with five implants (15.2%) in the MIPS group and one implant (3.3%) in the LITT-P group. The difference between the groups was not statistically significant [Kaplan–Meier analysis with log-rank test, the hazard ratio (95% confidence interval) = 4.71 (0.6–40.3) and *p* = 0.12]. The implant losses were registered between 26 and 99 days. In the MIPS group, three of five implants were lost spontaneously without previous signs of inflammation or pain, one implant was extruded after minor trauma, and one implant was lost after an episode of recurrent soft tissue inflammation (Holgers ≥ 2, despite local and systemic antibiotic treatment). The extruded implant in the LITT-P group was also lost after recurrent soft tissue inflammation (despite local and systemic treatment). For four of the six extruded implants, there was a decline in ISQ measures in consecutive postoperative visits before implant loss. There was no relation between extrusion rate and abutment lengths (three abutments of 9 mm and also three abutments of 12 mm).

### Implant Stability Quotient

**Figure 3:**
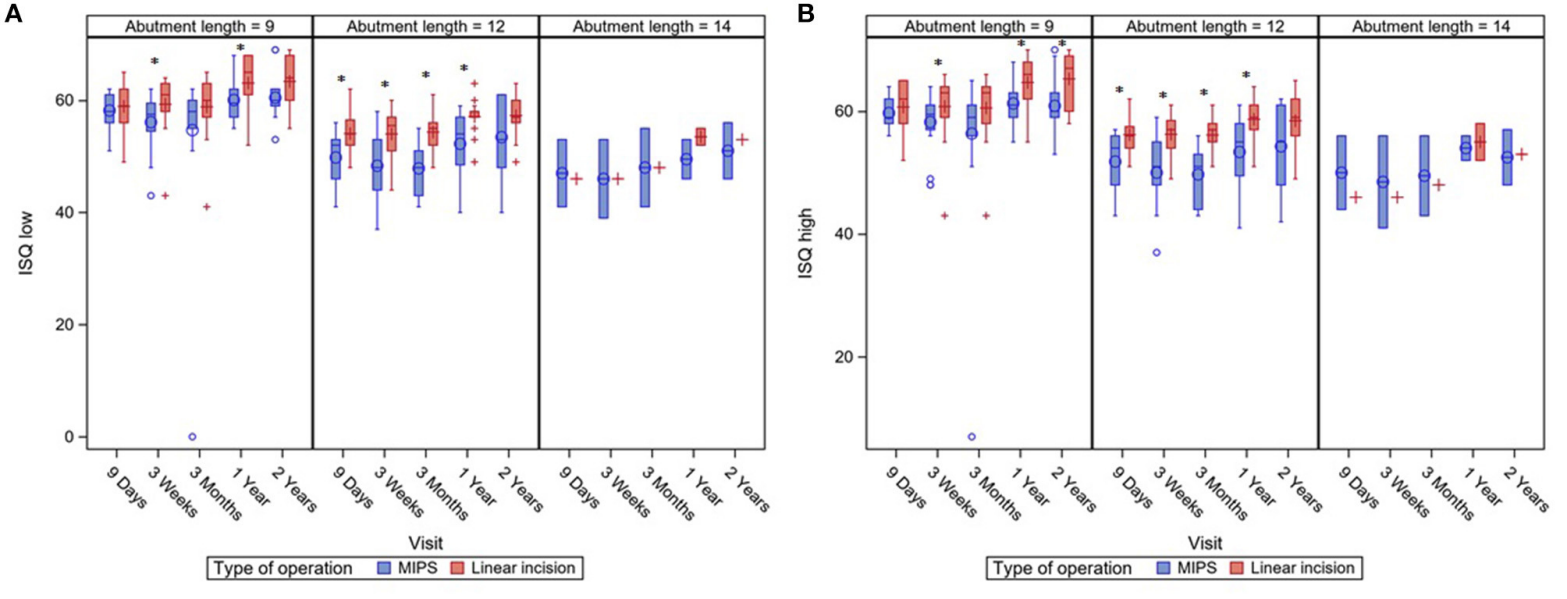
Boxplots of ISQ measurements during standard follow-up visits with a subdivision for different abutment lengths. ISQ measurements are displayed for ISQ Low **(A)** en ISQ High **(B)**. Asterisks (*) indicate a significant difference as calculated with a Mann–Whitney *U*-test (level of significance *p* < 0.05). Last visit (“2 years”) was at 22 months of follow-up.

No significant differences in ISQ low and high after implantation of the BCD were seen between the two different surgical approaches. An additional analysis, correcting for abutment length, showed that LITT-P patients had significantly higher ISQ low and ISQ high over time compared with patients after the MIPS procedure (Figure 3, Supplementary Figure 2).

### Tertiary Outcomes

The questionnaires HUI-III, APHAB, and ICECAP-A were used to assess the impact on hearing specific and general quality of life and capabilities (Supplementary Figure 3). In the HUI-III, the subjects who underwent the LITT-P had a significantly better single attribution score for pain (at 12- and 22-month follow-up and compared with the preoperative baseline at 22-month follow-up) and a lower score for vision (compared with preoperative baseline at 12- and 22-month follow-up). No differences in overall sum score and other single attribution scores were found between the groups during follow-up. The APHAB and ICECAP-A did not reveal any significant difference in respectively global score (or any subdomains) and tariff value (or any life domains) between the MIPS and LITT-P cohort. An overview of the results at 22-month follow-up is shown in Supplementary Figure 3.

## Discussion

In this multicenter randomized controlled trial, a comparison of the surgical outcomes is made between the MIPS technique and the LITT-P approach with a follow-up of 22 months. There were no differences in the presence of (adverse) soft tissue reactions between these techniques during complete follow-up. Also, no differences in pain related to the implant, wound dehiscence, mean skin level, soft tissue overgrowth, and overall quality of life were found between the groups. However, the outcomes for loss of sensibility (until 3-month post-surgery), cosmetic scores, and skin sagging were better in the MIPS cohort. Furthermore, the ISQ was higher in the LITT-P cohort for different abutment lengths at various times during follow-up. Finally, a nonsignificantly higher rate of implant extrusion was found after the MIPS procedure compared with the LITT-P procedure (15.2 vs. 3.3%).

Over the past few years, the punch-only techniques have been developed because of the minimally invasive nature of the surgery with possibly associated benefits. The short-term results were promising with improved outcomes on cosmetic appearance, skin sagging, sensibility loss, and surgical time without registration of more soft tissue problems in comparison with other current implantation techniques. Nevertheless, implant extrusion was mentioned several times as a warrant for further research (29–33, 35, 39). Also, there were limited long-term results with only one small cohort study (36). Moreover, a standardized procedure was lacking. In this context, the MIPS provided a clear, structured procedure, including a surgical kit (33). To our knowledge, this is the first multicenter randomized controlled trial using the standardized MIPS with a long-term follow-up. Other strengths of this study are the large sample size (*n* = 63), no differences in implant type between the groups, and combining both relevant clinical and patient-related outcomes (including objective and subjective measures). Also, the strict registration of adverse events conform to protocol during both standard and extra visits, could lead to a more reliable reflection of (the number of) complications.

The primary outcome measure of this study is soft tissue reaction. There is a relatively higher prevalence of adverse soft tissue reactions and Holgers grade 3 for both techniques in this study in comparison with previous studies into tissue preservation techniques [for review, see Verheij et al. (7)]. Possible explanations can be the relatively long follow-up of this study, the strict adherence to the protocol, and also the interobserver variability of the Holgers index (6). Regarding the other soft tissue outcomes, there was less skin sagging after the MIPS operation compared with after LITT-P, possibly as a result of less soft tissue mobilization during MIPS surgery. Also, the extent of skin sagging may be influenced by soft tissue manipulation after the LITT-P procedure due to placement of the implant slightly lateral to the incision. Nonetheless, the adverse soft tissue reactions, maximum Holgers index, wound dehiscence, mean skin level, and soft tissue overgrowth did not differ between both techniques.

The pain scores showed no differences between the techniques and were generally low in this study. However, the single attribution score for pain in the quality of life questionnaire HUI-III was significantly better in the LITT-P cohort. The much broader definition of pain could explain this discrepancy in the context of quality of life. Because the pain scores related to the BCD (as judged by the patients) are generally low and not different between both techniques, it is not likely that this difference in the HUI- III could be attributed to the surgical technique. Finally, it is relevant to mention in this context that more invasive implantation techniques, such as the LITT-R and Dermatome technique, also have favorable pain scores (i.e., most patients experience no or only mild pain) (8, 22).

Although sensibility loss was less in the MIPS cohort until 3 months of follow-up, no difference in sensibility loss was found at long-term follow-up. This can be related to the improvement of cutaneous sensibility after 1-year post-surgery, which is in accordance with other surgeries, such as otoplasty (40). Similar results have been reported in another study comparing MIPS with LITT-PP, where there was a tendency for better sensibility outcome after surgery and with the comparable outcome at 6 months; however, in subjective numbness, the MIPS technique was significantly better (39). Potentially, this could reflect a process of regenerating (sensible) nerve units. The cosmetic outcomes as assessed by the surgeon were relatively favorable in both techniques; however, the results were better in the MIPS cohort. This is in line with the study of Caspers et al. (39). Nevertheless, although there were these differences, patient satisfaction of the cosmetic results with (or without) processor did not differ between the groups. This leads to the discussion that one could argue about the relevance of the better cosmetic results (as scored by the surgeon) of the MIPS in the decision-making for one of the techniques.

The ISQ measure is used as an indicator for implant stability. Because abutment length is known to influence ISQ measures (41) (as also confirmed by our data), a subanalysis demonstrated significantly higher ISQ values in the LITT-P cohort for different abutment lengths at various time points during follow-up compared with the MIPS cohort. In the current literature, the clinical relevance of the difference in ISQ value and its link to osseointegration are being discussed (41, 42). The individual absolute ISQ values for a particular implant system, leading to success or failure, are unknown. However, the trend in ISQ over time might be indicative of implant-bone stability for an individual implant (41). Nevertheless, the usefulness of individual ISQ measures is still unknown (41). Moreover, specifically regarding this study, previous literature did observe already lower ISQ values after MIPS surgery, which has been associated with the slightly different osteotomy shape (43). Nonetheless, the indication of better osseointegration with these higher ISQ values after the LITT-P cannot be excluded. In addition, although statistically nonsignificant, there was more implant loss in the MIPS cohort. Less or delayed osseointegration as a possible factor could also not be excluded and might explain the rate of implant loss in our MIPS cohort, which is relatively high compared with previous studies (5, 7, 44).

Other explanations for nonsignificantly more implant extrusions after the MIPS compared with LITT-P in this study can be postulated. First of all, the visibility at the implant site during surgery is reduced for MIPS compared with an open approach. This lack of exposure can lead to incomplete and/or angulated insertion (30, 35). Secondly, the smaller incision and the guided drill approach may result in reduced access for external irrigation to the osteotomy. Inadequate irrigation is a risk for excessive heat generation during drilling (35, 45). Previous researches, mainly in dental surgery, have shown that heat generation negatively affects the bone/osseointegration at the implant site. Depending on the amount of heat generated, it is possible that the bony turnover can be impaired due to necrosis, osteocytic degeneration, fibrosis, and increased osteoclastic activity. Besides external irrigation, there are different other factors influencing heat generation: operator (e.g., pressure, speed, and duration of drilling), equipment (e.g., design and sharpness of the drill), and patient-related facets (e.g., age, bone density, and implant location) (45, 46). Possible solutions for this issue of heat generation may be improved drill systems that allow for better irrigation (including alteration in the shape of the drill and/or in the field of irrigation with the addition of internal irrigation). Also, the awareness of surgeons that the increase in temperature is directly proportional to the duration of drilling is important (46, 47).

In a recent bench study, the heat generation when drilling in artificial bone with the MIPS drill system and the conventional system for an open approach were compared (47). The study confirmed that for both systems, when used according to the recommended and uncompromised clinical protocol, the heat generation was below the threshold for thermally induced damage. Interestingly, the study revealed that the MIPS system was less sensitive to a reduction of the irrigation, whereas it was much more sensitive to a prolonged drilling procedure, indicating an important contribution of the operator performing the drilling procedure (47).

A third explanation of the impaired osseointegration might be soft tissue entering the drill hole despite using a cannula, which may lead to entrapment of soft tissue fragments in the osteotomy when inserting the implant. These hypotheses mentioned earlier may imply the need for advanced clinical experience when using the MIPS approach. In correspondence with the findings using flapless dental implant placement techniques, there seems to be a learning curve to achieve treatment success. A learning curve for the MIPS technique could potentially also be a prerequisite for adequate implantation (48–50). For example, surgeons with more experience in BCD surgery may encounter fewer difficulties with limited vision during implantation. Also, a more routine and faster procedure will reduce the drilling time and thus thermal damage to the implant site. Training and adherence to the instructions and cautions seem relevant for the success of the procedure. Although this was stated already previously (33, 35), the present study did not find any learning effects based on our adverse events log, potentially resulting from including only experienced surgeons.

Finally, the quality of life was assessed in this study with not only a hearing-specific questionnaire (APHAB) (51) but also a health status classification questionnaire (HUI- III) (52) and a capability measure (ICECAP-A) (53). There were no differences in overall quality of life between both cohorts during follow-up. This multi-domain evaluation of the quality of life is an extra-strength of this study because it tells us something about the impact of our primary and secondary outcomes on a patient's functioning in their daily life. In fact, the relevance of the primary and secondary results for patients might be discussed if the quality of life between the groups does not differ. This could mean that the differences found in clinical outcomes may not be important factors in the opinion of the patients in the population. On the other hand, one might argue about the validity of the questionnaires for this intervention. Perhaps, more procedure-related questionnaires might be more sensitive.

Although the various strengths of the current study, the limitations of this study should be considered when interpreting the results. Firstly, all surgeons had a long time of experience with the linear incision technique. In contrast, the MIPS is a relatively new technique with a learning curve. However, as we did not find any learning effects, it is unlikely that this influenced our data. Secondly, some outcome measures regarding soft tissue problems and cosmetics imply an interobserver variability, which could have influenced the results. The fact that the surgeons and researcher could not be blinded might attribute to this point. Finally, as already described in the 3-month follow-up results (32), the study population was of Caucasian origin. It has previously been indicated that the risk of soft tissue problems after BCD surgery (particularly scar formation) is higher in the African-American population (54).

In conclusion, these long-term results show favorable outcomes for both techniques regarding soft tissue reactions, pain, patient satisfaction, and quality of life. The MIPS has better outcomes in the context of skin sensibility (on short-term results), cosmetic appearance, and skin sagging in comparison with the LITT-P. In combination with the previously described significantly shorter surgical time (35), MIPS is a promising technique. Nevertheless, as demonstrated in the discussion, the results show concerns regarding osseointegration and implant extrusion after the MIPS procedure. Possibly, this might be explained by less exposure during the procedure with more risk on angulated insertion, prolonged drilling time, inadequate irrigation, and the need for gaining surgical experience. However, after 3 months of follow-up, no implants were lost. Future developments in irrigation, drilling systems, and optimized standardized surgical procedures and training may overcome these problems and should be a focus for further research.

## Data Availability Statement

The raw data supporting the conclusions of this article will be made available by the authors, without undue reservation.

## Ethics Statement

The studies involving human participants were reviewed and approved by There was approval by the ethics committee at Maastricht University Medical Centre+ (NL500720.068.14), Medisch Centrum Leeuwarden and Ziekenhuisgroep Twente. Also, there was registration in ClinicalTrials.gov NCT02438618. The patients/participants provided their written informed consent to participate in this study.

## Author Contributions

All listed authors made a significant contribution in the conception, design and formation of the manuscript/study. Moreover, all authors did revise the work and approve this final version. RS, LS, TC, AB, HB, MW, EE, JT, RS and MLJ had an important role in the data collection/acquisition. RS, LS, TC, MLJ, AB, SJ, MH and RS contributed in the data analysis. All authors contributed to the article and approved the submitted version.

## Conflict of Interest

MLJ, SJ, and MH are paid employees of Oticon Medical. The remaining authors declare that the research was conducted in the absence of any commercial or financial relationships that could be construed as a potential conflict of interest.

## Supplementary Material

Supplementary Figure 1 Stacked bar chart for the highest observed Holgers Index scores during standard follow-up visits (PP analysis). The last visit (“2 years”) was at 22 months of follow-up.

Supplementary Figure 2 Boxplots of ISQ measurements during standard follow-up visits with a subdivision for different abutment lengths (PP population). ISQ measurements are displayed for ISQ Low **(A)** en ISQ High **(B)**. An asterisks (^*^) indicates a significant difference as calculated with a Mann-Whitney *U*-test (level of significance *p* < 0.05). The last visit (“2 years”) was at 22 months of follow-up.

Supplementary Figure 3 Results of the quality of life questionnaires at 22 months follow-up (with 95% confidence intervals). An asterisks (^*^) indicates a significant difference as calculated with a Mann-Whitney *U*-test (level of significance *p* < 0.05). **(A)** The Abbreviated Profile of Hearing Aid Benefit (APHAB) including the four subscales (with or without BCD). The scale “Aversiveness” means the unpleasantness of environmental sounds. “Reverberation” indicates the communication in reverberant surroundings. “Background noise” specifies the communication in spaces with high background noise levels. The sub-item “Ease of communication” means the strain of communication under relatively favorable conditions. Finally, the “global score” is calculated as the mean score of the subscales reverberation, background noise and ease of communication. The displayed mean score can vary between 1 and 99% which show how frequent the subjects experience difficulties in (this field of) hearing performance. **(B)** The Health Utilities Index Mark III (HUI-III) including the single attribute utility functions. The displayed mean scores range from 0 to 1 with 1.00 indicating a perfect health status. The overall (utility) score is calculated based on the different weighted single attribute utility scores. **(C)** The ICEpop CAPability measure for Adults (ICECAP-A) which compromises five domains of capabilities: “enjoyement and pleasure” (an ability to experience enjoyment and pleasure), “achievement and progress” (an ability to achieve and progress in life), “feeling settled” (an ability to feel settled and secure), “love and friendship” (an ability to have love, friendship and support), and “being independent” (an ability to be autonomous). The mean scores are presented with the “top” level (full capability) takes the value “4” and the bottom level (no capability) takes the value “1”. Finally, the tariff value is calculated based on the different weighted individual attributes. This is an overall state of capability and ranges between 0 and 1 (with “1” as highest score for overall capability).

Supplementary Table 1 Baseline characteristics per protocol population.

Supplementary Table 2 Primary and secondary outcomes (PP population). Continuous variables are presented as mean (SD, 95% CI) and median (min–max). The *n* is showed at 22 months follow-up visits and otherwise if it differs from the PP population numbers. A significant *p*-value (*p* < 0.05) is showed in bold. Pain is graded in a 10-point scale with a scale of 0 representing absence of pain to 10 representing the worst pain. The area of sensibility loss is registered as the most outward diameter from abutment (in mm). The cosmetic observed variables are rated as 1 being no difference with the healthy contralateral site and with 10 being the most negative difference with the healthy situation. Only the overall cosmetic score and patient satisfaction will be rated with 10 being the best cosmetic result. The skin level is measured as the distance between the top of the abutment to the skin in four quadrants (in mm).
