# Supplementary figures and images for: Long-Term Outcomes of the Minimally Invasive Ponto Surgery vs. Linear Incision Technique With Soft Tissue Preservation for Installation of Percutaneous Bone Conduction Devices
Source: Front Neurol. 2021 Feb 24;12:632987. doi: 10.3389/fneur.2021.632987 (PMC7945693; doi:10.3389/fneur.2021.632987)

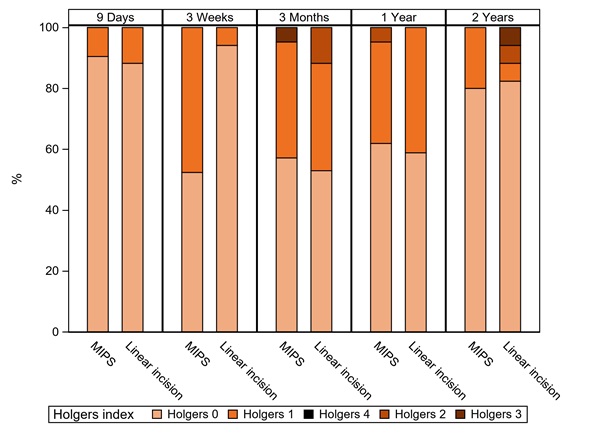

Supplement: Supplementary Figure 1 — Stacked bar chart for the highest observed Holgers Index scores during standard follow-up visits (PP analysis). The last visit (“2 years”) was at 22 months of follow-up. [file Image_1.JPEG]

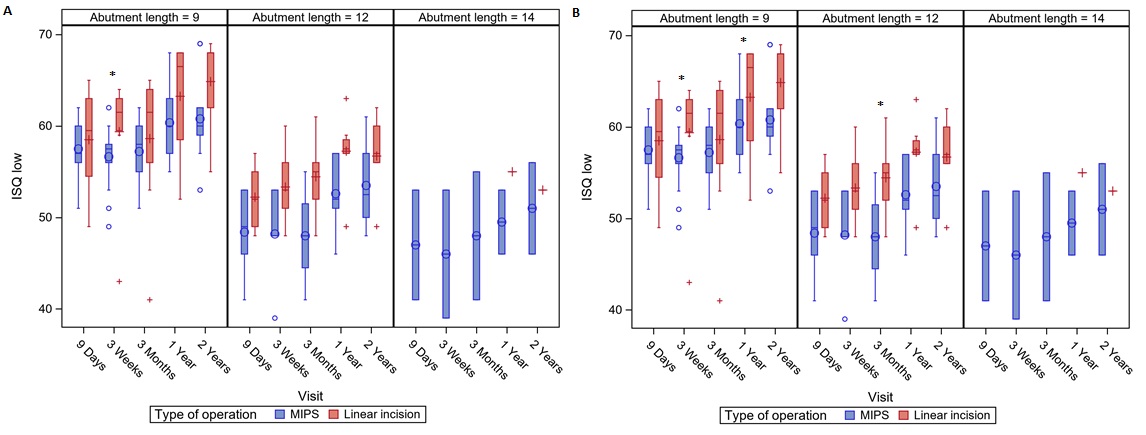

Supplement: Supplementary Figure 2 — Boxplots of ISQ measurements during standard follow-up visits with a subdivision for different abutment lengths (PP population). ISQ measurements are displayed for ISQ Low (A) en ISQ High (B). An asterisks (*) indicates a significant difference as calculated with a Mann-Whitney U-test (level of significance p < 0.05). The last visit (“2 years”) was at 22 months of follow-up. [file Image_2.JPEG]

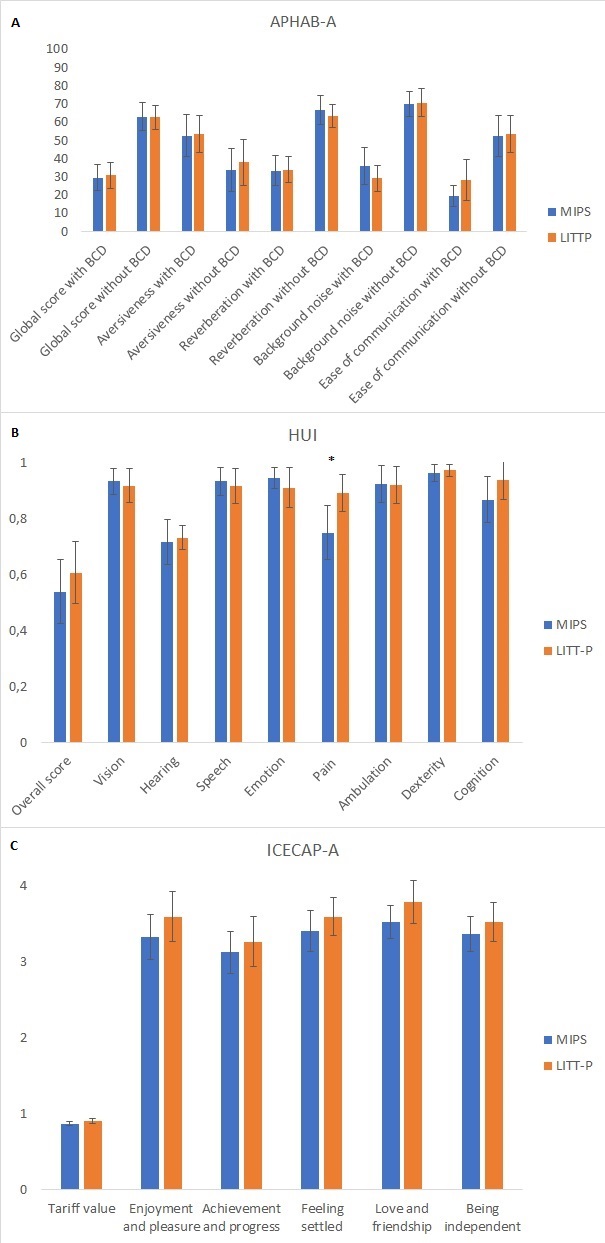

Supplement: Supplementary Figure 3 — Results of the quality of life questionnaires at 22 months follow-up (with 95% confidence intervals). An asterisks (*) indicates a significant difference as calculated with a Mann-Whitney U-test (level of significance p < 0.05). (A) The Abbreviated Profile of Hearing Aid Benefit (APHAB) including the four subscales (with or without BCD). The scale “Aversiveness” means the unpleasantness of environmental sounds. “Reverberation” indicates the communication in reverberant surroundings. “Background noise” specifies the communication in spaces with high background noise levels. The sub-item “Ease of communication” means the strain of communication under relatively favorable conditions. Finally, the “global score” is calculated as the mean score of the subscales reverberation, background noise and ease of communication. The displayed mean score can vary between 1 and 99% which show how frequent the subjects experience difficulties in (this field of) hearing performance. (B) The Health Utilities Index Mark III (HUI-III) including the single attribute utility functions. The displayed mean scores range from 0 to 1 with 1.00 indicating a perfect health status. The overall (utility) score is calculated based on the different weighted single attribute utility scores. (C) The ICEpop CAPability measure for Adults (ICECAP-A) which compromises five domains of capabilities: “enjoyement and pleasure” (an ability to experience enjoyment and pleasure), “achievement and progress” (an ability to achieve and progress in life), “feeling settled” (an ability to feel settled and secure), “love and friendship” (an ability to have love, friendship and support), and “being independent” (an ability to be autonomous). The mean scores are presented with the “top” level (full capability) takes the value “4” and the bottom level (no capability) takes the value “1”. Finally, the tariff value is calculated based on the different weighted individual attributes. This is an overall state of capability an [file Image_3.JPEG]
