# Supplementary material for: Long-Term Outcomes of the Minimally Invasive Ponto Surgery vs. Linear Incision Technique With Soft Tissue Preservation for Installation of Percutaneous Bone Conduction Devices
Source: Front Neurol. 2021 Feb 24;12:632987. doi: 10.3389/fneur.2021.632987 (PMC7945693; doi:10.3389/fneur.2021.632987)
Supplement: Supplementary Table 1 — Baseline characteristics per protocol population. [file Table_1.DOCX]

| **Baseline characteristic** | **MIPS (n = 21)** | **LITT-P (n = 17)** | ***p*-value** |
| --- | --- | --- | --- |
| Age (years) |  |  |  |
| Mean (SD), 95 % - CI | 52.1 (15.1), 45.3; 59.0 | 51.0 (17.3), 42.1; 59.9 | 0.83 |
| Median (Min - Max) | 53.0 (30.0 - 80.0) | 52.0 (24.0 - 75.0) |  |
| Gender |  |  |  |
| Male | 8 (38.1 %) | 7 (41.2 %) | 1.00 |
| Female | 13 (61.9 %) | 10 (58.8 %) |  |
| Type of hearing losse |  |  |  |
| Acquired conductive/mixed hearing loss | 16 (76.2 %) | 16 (94.1 %) | 0.30 |
| Single sided deafness | 4 (19.0 %) | 1 (5.9 %) |  |
| Congenital conductive hearing loss | 1 (4.8 %) | 0 (0.0 %) |  |
| Smoking |  |  |  |
| Yes | 3 (14.3 %) | 4 (23.5 %) | 0.75 |
| No | 18 (85.7 %) | 13 (76.5 %) |  |
| Body Mass Index (BMI; kg/m2) |  |  |  |
| Mean (SD), 95 % - CI | 27.7 (6.5), 24.7; 30.7 | 28.1 (4.7), 25.6; 30.7 | 0.62 |
| Median (Min - Max) | 27.2 (20.5 - 44.4) | 27.0 (22.0 - 36.3) |  |
| Ethinicity |  |  |  |
| Caucasian | 21 (100.0 %) | 17 (100.0 %) |  |
| Implantation site |  |  |  |
| Right | 13 (61.9 %) | 7 (41.2 %) | 0.34 |
| Left | 8 (38.1 %) | 10 (58.8 %) |  |
| Abutment length |  |  |  |
| 9 | 14 (66.7 %) | 8 (47.1 %) | 0.224 |
| 12 | 5 (23.8 %) | 9 (52.9 %) | 0.064 |
| 14 | 2 (9.5 %) | 0 (0.0 %) |  |

**Supplementary table 1:** Baseline characteristics per protocol population.
