# Supplementary material for: Long-Term Outcomes of the Minimally Invasive Ponto Surgery vs. Linear Incision Technique With Soft Tissue Preservation for Installation of Percutaneous Bone Conduction Devices
Source: Front Neurol. 2021 Feb 24;12:632987. doi: 10.3389/fneur.2021.632987 (PMC7945693; doi:10.3389/fneur.2021.632987)
Supplement: Supplementary Table 2 — Primary and secondary outcomes (PP population). Continuous variables are presented as mean (SD, 95% CI) and median (min–max). The n is showed at 22 months follow-up visits and otherwise if it differs from the PP population numbers. A significant p-value (p < 0.05) is showed in bold. Pain is graded in a 10-point scale with a scale of 0 representing absence of pain to 10 representing the worst pain. The area of sensibility loss is registered as the most outward diameter from abutment (in mm). The cosmetic observed variables are rated as 1 being no difference with the healthy contralateral site and with 10 being the most negative difference with the healthy situation. Only the overall cosmetic score and patient satisfaction will be rated with 10 being the best cosmetic result. The skin level is measured as the distance between the top of the abutment to the skin in four quadrants (in mm). [file Table_2.docx]

|  |  |  |  |  |
| --- | --- | --- | --- | --- |
| **Primary outcome: (adverse) soft tissue reactions** |  | **MIPS (n = 21)** | **LITT-P (n = 17)** | ***p*-value** |
| Adverse soft tissue reaction (Holgers ≥ 2) from surgery to 22months |  | 5 (23.8 %) | 8 (47.1 %) | 0.13 |
| Adverse soft tissue reaction (Holgers ≥ 2) from surgery to 22 months (with the Fisher's Exact Test) |  | 5 (23.8 %) | 8 (47.1 %) | 0.25 |
| Maximum Holgers Index at standard and extra visits |  |  |  |  |
| 0 No irritation |  | 6 (28.6 %) | 1 (5.9 %) | 0.20 |
| 1 Slight redness |  | 10 (47.6 %) | 8 (47.1 %) |  |
| 2 Red and slightly moist tissue |  | 2 (9.5 %) | 7 (41.2 %) |  |
| 3 Reddish and moist tissue, sometimes granulation formation |  | 3 (14.3 %) | 1 (5.9 %) |  |
| 4 Profound signs of infection resulting in implant removal |  | 0 (0.0 %) | 0 (0.0 %) |  |
| **Secondary outcome: pain** |  |  |  |  |
| Pain around the implant |  |  |  |  |
| 9 days |  | 1.71 (2.05, 0.78;2.65) 1.00 (0.00 - 6.00) | 2.41 (2.72, 1.01;3.81) 2.00 ( 0.00 - 8.00) | 0.43 |
| 3 weeks |  | 0.952 (1.284, 0.368;1.537) 0.00 (0.00 - 4.00) | 0.647 (1.115, 0.074;1.220) 0.00 (0.00 -3.00 | 0.34 |
| 3 months |  | 1.52 (2.02, 0.61;2.44) 0.00 (0.00 - 6.00) | 0.941 (1.638, 0.099;1.1783) 0.00 (0.00 - 5.00) | 0.24 |
| 12 months |  | 0.952 (1.936, 0.071;1.834) 0.00 (0.00 - 6.00) | 1.53 (2.48, 0.26;2.80) 0.00 (0.00 - 8.00) | 0.44 |
| 22 months |  | 0.400 (0.821, 0.016;0.784) 0.00 (0.00 - 2.00) n = 20 | 0.941 (1.749, 0.042;1.840) 0.00 (0.00 - 5.00) n = 17 | 0.43 |
| Radiating pain from the implant |  |  |  |  |
| 9 days |  | 0.810 (1.965, -0.085;1.704) 0.00 (0.00 - 7.00) | 0.824 (2.038, -0.224;1.871) 0.00 (0.00 - 8.00) | 0.80 |
| 3 weeks |  | 0.667 (1.592, -0.058;1.391) 0.00 (0.00 - 5.00) | 0.176 (0.728, -0.198;0.551) 0.00 (0.00 - 3.00) | 0.24 |
| 3 months |  | 0.762 (1.814, -0.064;1.588) 0.00 (0.00 - 6.00) | 0.529 (1.505, -0.244;1.303) 0.00 (0.00 -5 .00) | 0.39 |
| 12 months |  | 0.381 (1.244, -0.185;0.947) 0.00 (0.00 - 5.00) | 0.294 (1.213, -0.329;0.918) 0.00 (0.00 - 5.00) | 0.73 |
| 22 months |  | 0.200 (0.894, -0.219;0.619) 0.00 (0.00 - 4.00) n = 20 | 0.294 (0.849, -0.142;0.731) 0.00 (0.00 - 3.00) n =17 | 0.52 |
| Headache related to the BCD |  |  |  |  |
| 9 days |  | 0.667 (1.713, -0.113;1.446) 0.00 (0.00 - 7.00) | 1.29 (2.28, 0.12;2.47) 0.00 (0.00 - 7.00) | 0.40 |
| 3 weeks |  | 0.571 (1.805, -0.250;1.393) 0.00 (0.00 - 6.00) | 0.118 (0.485, -0.132;0.367) 0.00 (0.00 -2.00) | 0.66 |
| 3 months |  | 0.905 (2.322, -0.152;1.962) 0.00 (0.00 - 8.00) | 0.412 (1.064, -0.135;0.959) 0.00 (0.00 - 4.00) | 0.96 |
| 12 months |  | 0.381 (1.359, -0.238;1.000) 0.00 (0.00 - 6.00) | 0.00 (0.00, 0.00;0.00) 0.00 (0.00 - 0.00) | 0.21 |
| 22 months |  | 0.00 (0.00, 0.00;0.00) 0.00 (0.00 - 0.00) n = 20 | 0.412 (1.698, -0.461;1.285) 0.00 (0.00 - 7.00) n = 17 | 0.30 |
| **Secondary outcome: sensibility** |  |  |  |  |
| Area loss of sensibility (most outward diameter from abutment in mm) |  |  |  |  |
| 9 days |  | 3.67 (7.47, 0.27;7.07) 0.00 (0.00 - 25.00) | 12.8 (14.6, 5.3;20.3) 15.0 (0.0 - 50.0) | **0.022** |
| 3 weeks |  | 0.333 (1.155, -0.192;0.859) 0.00 (0.00 - 5.00) | 4.24 (10.15, -0.98;9.46) 0.00 (0.00 - 35.00) | 0.21 |
| 3 months |  | 0.095 (0.436, -0.103;0.294) 0.00 (0.00 - 2.00) | 2.82 (6.61, -0.57;6.22) 0.00 (0.00 - 25.00) | 0.080 |
| 12 months |  | 0.048 (0.218, -0.052;0.147) 0.00 (0.00 - 1.00) | 1.71 (4.84, -0.79;4.20) 0,00 (0.00 - 18.00) | 0.20 |
| 22 months |  | 1.18 (4.85, -1.32;3.67) 0.00 (0.00 - 20.00) n = 17 | 0.00 (0.00, 0.00;0.00) 0.00 (0.00 - 0.00) n = 14 | 0.40 |
| Presence of loss of sensibility? |  |  |  |  |
| 9 days | No | 16 (76.2 %) | 7 (41.2 %) | 0.062 |
|  | Yes | 5 (23.8 %) | 10 (58.8 %) |  |
| 3 weeks | No | 19 (90.5 %) | 13 (76.5 %) | 0.47 |
|  | Yes | 2 (9.5 %) | 4 (23.5 %) |  |
| 3 months | No | 20 (95.2 %) | 13 (76.5 %) | 0.22 |
|  | Yes | 1 (4.8 %) | 4 (23.5 %) |  |
| 12 months | No | 20 (95.2 %) | 14 (82.4 %) | 0.45 |
|  | Yes | 1 (4.8 %) | 3 (17.6 %) |  |
| 22 months | No | 16 (94.1 %) | 14 (100.0 %) | 1.00 |
|  | Yes | 1 (5.9 %) | 0 (0.0 %) |  |
| **Secondary outcome: cosmetic appearance** |  |  |  |  |
| Natural skin position |  |  |  |  |
| 3 months |  | 2.81 (1.12, 2.30;3.32) 3.00 (1.00 - 5.00) | 3.71 (1.40, 2.98;4.43) 3.00 (1.00 - 6.00) | **0.048** |
| 12 months |  | 2.14 (1.15, 1.62;2.67) 2.00 (1.00 - 5.00) | 3.06 (1.25, 2.42;3.70) 3.00 (1.00 - 5.00) | **0.014** |
| 22 months |  | 1.94 (1.66, 1.12;2.77) 1.50 (1.00 - 8.00) n = 18 | 2.50 (1.87, 1.42;3.58) 2.00 (1.00 - 7.00) n = 14 | 0.28 |
| Extent of baldness |  |  |  |  |
| 3 months |  | 2.24 (0.83, 1.86;2.62) 2.00 (1.00 - 4.00) | 4.00 (1.32, 3.32;4.68) 4.00 (1.00 - 6.00) | **<.0001** |
| 12 months |  | 1.95 (0.92, 1.53;2.37) 2.00 (1.00 - 4.00) n = 21 | 3.25 (1.34, 2.54;3.96) 3.50 (1.00 - 5.00) n = 16 | **0.0037** |
| 22 months |  | 1.94 (1.95, 0.97;2.92) 1.00 (1.00 - 9.00) n = 18 | 2.21 (1.12, 1.57;2.86) 2.00 (1.00 - 4.00) n = 14 | 0.15 |
| Scarring |  |  |  |  |
| 3 months |  | 2.38 (1.02, 1.92;2.85) 2.00 (1.00 - 5.00) | 4.94 (1.60, 4.12;5.76) 5.00 (1.00 - 7.00) | **<.0001** |
| 12 months |  | 2.14 (1.24, 1.58;2.71) 2.00 (1.00 - 5.00) | 4.06 (1.60, 3.24;4.88) 4.00 (1.00 - 7.00) | **0.0006** |
| 22 months |  | 2.28 (1.87, 1.35;3.21) 2.00 (1.00 - 9.00) n = 18 | 2.29 (1.20, 1.59;2.98) 2.00 (1.00 - 5.00) n = 14 | 0.57 |
| Skin colour |  |  |  |  |
| 3 months |  | 3.10 (1.04, 2.62;3.57) 3.00 (1.00 - 5.00) | 4.24 (1.03, 3.70;4.77) 4.00 (3.00 - 6.00) | **0.0047** |
| 12 months |  | 2.48 (1.08, 1.99;2.97) 2.00 (1.00 - 4.00) | 3.71 (1.21, 3.08;4.33) 4.00 (2.00 - 6.00) | **0.0041** |
| 22 months |  | 2.11 (1.97, 1.13;3.09) 1.00 (1.00 - 9.00) n = 18 | 2.64 (1.28, 1.91;3.38) 2.00 (1.00 - 5.00) n = 14 | 0.065 |
| Indentation |  |  |  |  |
| 3 months |  | 2.24 (0.94, 1.81;2.67) 2.00 (1.00 - 5.00) | 4.12 (1.54, 3.33;4.91) 4.00 (1.00 - 7.00) | **0.0002** |
| 12 months |  | 2.24 (1.22, 1.68;2.79) 2.00 (1.00 - 5.00) | 3.76 (1.68, 2.90;4.63) 4.00 (1.00 - 7.00) | **0.0047** |
| 22 months |  | 2.06 (2.10, 1.01;3.10) 1.00 (1.00 - 9.00) n = 18 | 2.50 (1.99, 1.35;3.65) 2.00 (1.00 - 7.00) n = 14 | 0.26 |
| Overall cosmetic score |  |  |  |  |
| 3 months |  | 8.52 (0.75, 8.18;8.87) 8.00 (7.00 - 10.00) | 6.94 (1.09, 6.38;7.50) 7.00 (6.00 - 9.00) | **<.0001** |
| 12 months |  | 8.81 (0.93, 8.39;9.23) 9.00 (7.00 - 10.00) | 7.41 (1.06, 6.86; 7.96) 7.00 (5.00 - 9.00) | **0.0003** |
| 22 months |  | 8.67 (0.84, 8.25;9.08) 9.00 (7.00 - 10.00) n = 18 | 7.64 (1.08, 7.02;8.27) 8.00 (6.00 - 9.00) n =14 | **0.0078** |
| Satisfaction with result without processor |  |  |  |  |
| 3 months |  | 8.37 (1.57, 7.61;9.13) 9.00 (4.00 - 10.00) n =19 | 8.31 (1.45, 7.54;9.08) 8.00 (6.00 - 10.00) n = 16 | 0.80 |
| 12 months |  | 7.94 (1.55, 7.17;8.72) 8.00 (5.00 - 10.00) n = 18 | 8.53 (1.18, 7.92;9.14) 8.00 (6.00 - 10.00) n = 17 | 0.31 |
| 22 months |  | 8.82 (1.29, 8.16;9.48) 9.00 (6.00 - 10.00) n = 17 | 8.36 (0.93, 7.82;8.89) 8.00 (7.00 - 10.00) n = 14 | 0.19 |
| Satisfaction with result with processor |  |  |  |  |
| 3 months |  | 7.37 (2.34, 6.24;8.50) 8.00 (1.00 - 10.00) n = 19 | 7.71 (1.65, 6.86;8.55) 8.00 (5.00 - 10.00) n = 17 | 0.94 |
| 12 months |  | 7.39 (2.38, 6.21;8.57) 8.00 (3.00 - 10.00) n = 18 | 8.00 (1.70, 7.13;8.87) 8.00 (5.00 - 10.00) n = 17 | 0.55 |
| 22 months |  | 7.41 (2.53, 6.11;8.71) 8.00 (3.00 - 10.00) n = 17 | 7.23 (1.54, 6.30;8.16) 7.00 (4.00 - 10.00) n = 13 | 0.60 |
| **Secondary outcome: soft tissue** |  |  |  |  |
| Mean skin level |  |  |  |  |
| 9 days |  | 5.23 (1.15, 4.70;5.75) 5.75 (3.25 - 7.25) n = 21 | 5.67 (0.90, 5.19;6.15) 5.75 (4.25 - 7.25) n =16 | 0.25 |
| 3 weeks |  | 4.99 (1.19, 4.44;5.53) 5.00 (3.00 - 7.00) | 5.06 (0.88, 4.61;5.51) 5.00 (3.25 - 7.00) | 0.82 |
| 3 months |  | 5.31 (1.27, 4.73;5.89) 5.00 (3.00 - 8.00) | 5.24 (0.71, 4.87;5.60) 5.00 (4.00 - 5.60) | 0.98 |
| 12 months |  | 5.56 (1.49, 4.88;6.24) 5.50 (3.00 - 8.00) | 5.54 (1.01, 5.03 - 6.06) 5.50 (4.00 - 8.00) | 1.00 |
| 22 months |  | 5.56 (1.66, 4.79;6.34) 5.63 (3.00 - 9.00) n = 20 | 5.79 (1.15, 5.20;6.38) 5.75 (4.00 - 8.00) n = 17 | 0.62 |
| Skin sagging in any quadrant |  |  |  |  |
| 9 days |  | 4 (19.0 %) | 8 (50.0 %) | 0.10 |
| 3 weeks |  | 5 (23.8 %) | 15 (88.2 %) | **0.0002** |
| 3 months |  | 5 (23.8 %) | 13 (76.5 %) | **0.0031** |
| 12 months |  | 5 (23.8 %) | 11 (64.7 %) | **0.026** |
| 22 months |  | 4 (20.0 %) | 11 (64.7 %) | **0.014** |
| Wound dehiscence |  |  |  |  |
| 9 days |  | 12 (57.1 %) | 13 (76.5 %) | 0.37 |
| 3 weeks |  | 2 (9.5 %) | 2 (11.8 %) | 1.00 |
| 3 months |  | 0.0 (0.0 %) | 0.0 (0.0 %) | 1.00 |
| 12 months |  | 0.0 (0.0 %) | 0.0 (0.0 %) | 1.00 |
| 22 months |  | 0.0 (0.0 %) | 0.0 (0.0 %) | 1.00 |
| Soft tissue overgrowth |  |  |  |  |
| Abutment changes |  | 2 (9.5 %) | 2 (11.8 %) | 1.00 |
| Revision surgery |  | 1 (4.8 %) | 0 (0.0 %) | 1.00 |

**Supplementary table 2:** Primary and secondary outcomes (PP population). Continuous variables are presented as mean (SD, 95% CI) and median (min - max). The n is showed at 22 months follow-up visits and otherwise if it differs from the PP population numbers. A significant p-value (p < 0.05) is showed in bold.

Pain is graded in a 10-point scale with a scale of 0 representing absence of pain to 10 representing the worst pain. The area of sensibility loss is registered as the most outward diameter from abutment (in mm). The cosmetic observed variables are rated as 1 being no difference with the healthy contralateral site and with 10 being the most negative difference with the healthy situation. Only the overall cosmetic score and patient satisfaction will be rated with 10 being the best cosmetic result. The skin level is measured as the distance between the top of the abutment to the skin in four quadrants (in mm).
